# Metazoan Scc4 Homologs Link Sister Chromatid Cohesion to Cell and Axon Migration Guidance

**DOI:** 10.1371/journal.pbio.0040242

**Published:** 2006-07-04

**Authors:** Vlad C Seitan, Peter Banks, Steve Laval, Nazia A Majid, Dale Dorsett, Amer Rana, Jim Smith, Alex Bateman, Sanja Krpic, Arnd Hostert, Robert A Rollins, Hediye Erdjument-Bromage, Paul Tempst, Claire Y Benard, Siegfried Hekimi, Sarah F Newbury, Tom Strachan

**Affiliations:** **1**Institute of Human Genetics, University of Newcastle, Newcastle upon Tyne, United Kingdom; **2**Institute of Cell and Molecular Biosciences, University of Newcastle, Newcastle upon Tyne, United Kingdom; **3**Edward A. Doisy Department of Biochemistry and Molecular Biology, Saint Louis University School of Medicine, Saint Louis, Missouri, United States of America; **4**Wellcome Trust/Cancer Research UK Gurdon Institute and Department of Zoology, University of Cambridge, Cambridge, United Kingdom; **5**Wellcome Trust Sanger Institute, Hinxton, United Kingdom; **6**Erasmus Medical Center, University of Rotterdam, Rotterdam, Netherlands; **7**Weill Graduate School of Medical Sciences, Cornell Medical College, New York, New York, United States of America; **8**Molecular Biology Program, Memorial Sloan-Kettering Cancer Center, New York, New York, United States of America; **9**Department of Biology, McGill University, Montreal, Canada; Stowers Institute for Medical ResearchUnited States of America

## Abstract

Saccharomyces cerevisiae Scc2 binds Scc4 to form an essential complex that loads cohesin onto chromosomes. The prevalence of Scc2 orthologs in eukaryotes emphasizes a conserved role in regulating sister chromatid cohesion, but homologs of Scc4 have not hitherto been identified outside certain fungi. Some metazoan orthologs of Scc2 were initially identified as developmental gene regulators, such as *Drosophila* Nipped-B, a regulator of *cut* and *Ultrabithorax,* and delangin, a protein mutant in Cornelia de Lange syndrome. We show that delangin and Nipped-B bind previously unstudied human and fly orthologs of Caenorhabditis elegans MAU-2, a non-axis-specific guidance factor for migrating cells and axons. PSI-BLAST shows that Scc4 is evolutionarily related to metazoan MAU-2 sequences, with the greatest homology evident in a short N-terminal domain, and protein–protein interaction studies map the site of interaction between delangin and human MAU-2 to the N-terminal regions of both proteins. Short interfering RNA knockdown of human MAU-2 in HeLa cells resulted in precocious sister chromatid separation and in impaired loading of cohesin onto chromatin, indicating that it is functionally related to Scc4, and RNAi analyses show that MAU-2 regulates chromosome segregation in C. elegans embryos. Using antisense morpholino oligonucleotides to knock down Xenopus tropicalis delangin or MAU-2 in early embryos produced similar patterns of retarded growth and developmental defects. Our data show that sister chromatid cohesion in metazoans involves the formation of a complex similar to the Scc2-Scc4 interaction in the budding yeast. The very high degree of sequence conservation between Scc4 homologs in complex metazoans is consistent with increased selection pressure to conserve additional essential functions, such as regulation of cell and axon migration during development.

## Abstract 2

A complex previously found only in yeast is described in metazoa, where it functions both in chromatid cohesion and in migration during development.

## Introduction

Sister chromatid cohesion in eukaryotes occurs by a well-conserved mechanism that depends on cohesins, protein complexes that bind to multiple sites on chromosome arms but are highly enriched at centromeres. At the heart of cohesins are tripartite SMC-kleisin complexes that form ring-like structures (see [ 1, 2] for recent reviews). Two large SMC proteins, Smc1 and Smc3, form a V-shaped heterodimer with ABC-like ATPases at the tips (or heads) of the two arms. A connecting α-kleisin subunit (Scc1/Mcd1 in budding yeast) completes the ring; its N- and C-terminal sequences are linked to the ATPase heads of the Smc3 and Smc1 proteins, respectively.

The α-kleisin subunit can be hydrolyzed at anaphase by a specific protease, separase, enabling release of sister chromatids previously entrapped by centromeric cohesins. In association with the connecting kleisin subunit is a fourth cohesin subunit, Scc3 in budding yeast or SA1/SA2 in human cells. Similar SMC-kleisin complexes are the basis of the condensins that compact chromosomes in preparation for chromosome segregation. In one model for sister chromatid cohesion, the SMC-kleisin complexes are envisaged to form a ring-like structure that topologically entraps sister chromatids from the time of their generation, following DNA synthesis, up to their separation at anaphase [ 3– 5]. Another model has the rings interacting with one another and with one sister chromatid apiece in order to snap sister chromatids together [ 6, 7].

There has been increasing appreciation of wider functional roles for proteins that regulate sister chromatid cohesion and chromosome condensation (see [ 8]). Individual subunits of cohesin and condensin complexes have been implicated in gene regulation, and accessory proteins that facilitate how they work may also have diverse functions. Among several proteins that interact with cohesins are those that load previously assembled cohesins onto chromosomes. In *Saccharomyces cerevisiae,* the Scc2 and Scc4 proteins form a complex for this purpose. Both proteins are essential, and in *scc2* or *scc4* mutants cohesin complexes form normally, but they do not bind to centromeres or chromosome arms, resulting in precocious sister chromatid separation (PSCS) [ 9].

In *S. cerevisiae,* the loading of cohesin complexes occurs just before the initiation of DNA replication and at frequent intervals along the chromosomes—although shortly after being loaded cohesins appear to relocate to regions between convergent transcription units [ 10, 11]. Because hydrolysis of ATP bound to the Smc1/Smc3 heads is essential for cohesin loading, the function of Scc2-Scc4 has been suggested to stimulate the required ATP hydrolysis [ 12]. In more complex metazoan cells, loading of cohesins is mostly achieved in the G1 phase, although it can commence at the end of mitosis. In egg extracts from *Xenopus laevis,* the assembly of the pre-replication complex is required for loading of Scc2 onto the chromatin and for efficient cohesin loading [ 13, 14].

Consistent with its essential function in regulating sister chromatid cohesion, Scc2 has been well conserved during evolution, and orthologs can be identified in apparently all eukaryotes where there is sufficient sequence information. Scc2 and other well-studied fungal orthologs, including Schizosaccharomyces pombe Mis4 and Coprinus cinereus Rad9, are known to be involved in various aspects of chromosome function and double-strand DNA repair [ 15– 23]. Metazoan orthologs of Scc2 have recently been implicated in regulating sister chromatid cohesion, such as X. laevis XScc2, *Drosophila melanogaster* Nipped-B, and human delangin [ 13, 24, 25].

Some of the metazoan Scc2 orthologs were initially identified as developmental regulators. Nipped-B is known to regulate *Ultrabithorax* and *cut,* a homeobox gene important in wing and limb development, and is envisaged to facilitate interaction between the promoter and remote enhancers of such genes [ 24, 26]. Delangin, the product of the *NIPBL* ( *Nipped-B*-like) gene, is abnormally expressed in Cornelia de Lange syndrome, a rare congenital malformation that is characterized by growth and mental retardation, specific craniofacial and limb abnormalities, and abnormalities in a variety of other organs and tissues [ 27, 28] (see [ 29] for a recent review). The precise mechanism of action of Nipped-B and delangin in developmental gene regulation is uncertain. Nipped-B and cohesin subunits appear to have opposing effects on target gene expression leading to the idea that Nipped-B and orthologs (sometimes called adherins) also facilitate localized or temporary unloading of cohesin to allow long range gene activation [ 24, 30, 31].

The structures of Scc2 orthologs provide few clues as to how they function in cells. A large C-terminal domain, spanning about 1,300–1,500 amino acids in the different family members, is comparatively strongly conserved and is expected to be functionally significant. The vast majority of CdLS-associated missense mutations map to this region in delangin (see [ 29]), and the domain contains several HEAT repeats, motifs that have been implicated in protein–protein interaction. HEAT repeats have been found in a variety of chromosome-associated proteins, including several other classes of proteins associated with cohesins and condensins [ 32, 33]. The N-terminal region of Scc2 orthlogs has expanded in size during evolution, but is comparatively poorly conserved.

Scc2 has been shown to physically interact with cohesin [ 12], but the wider (evolutionary) significance of its interaction with Scc4 has been enigmatic. In contrast to Scc2, Scc4 appears at first sight to have been very poorly conserved during evolution [ 9]. Using a Scc4 protein query to screen protein and translated nucleotide sequence databases, standard BLAST analyses identify significant sequence matches in a very restricted group of organisms (see below). Other than Scc4, only a few proteins have been reported to interact with members of the Scc2 protein family. Mammalian HP1 heterochromatin proteins have been shown to bind to delangin sequences, but the interaction motif in the delangin sequence is located in a comparatively poorly conserved N-terminal region [ 34, 35].

In order to explore the function of the Nipped-B and delangin developmental regulators, we sought proteins that interact with them. In the current study we show that Nipped-B and delangin interact respectively with the products of the *D. melanogaster CG4203* and human *KIAA0892* genes. The latter two genes have not previously been studied, but they have recently been recognized to be orthologs of *mau-2,* a gene that acts very early during development and that encodes a factor known to be important in guiding the migration of cells and axons during development [ 36, 37]. We have carried out PSI-BLAST analyses that show that MAU-2 and its metazoan orthologs are evolutionarily related to yeast Scc4. We also present evidence that the human MAU-2 protein is functionally, as well as evolutionarily, related to Scc4, and that MAU-2 regulates chromosome segregation in *Caenorhabditis elegans.* Finally, we show that Xenopus tropicalis MAU-2 and delangin are involved in similar developmental pathways. The data obtained indicate that metazoan Scc4 homologs provide an unanticipated link between sister chromatid cohesion and the guidance of cell and axon migration.

## Results

### Homologs of the C. elegans Cell and Axon Migration Guidance Factor MAU-2 Bind to Nipped-B and Delangin

**Figure 1:**
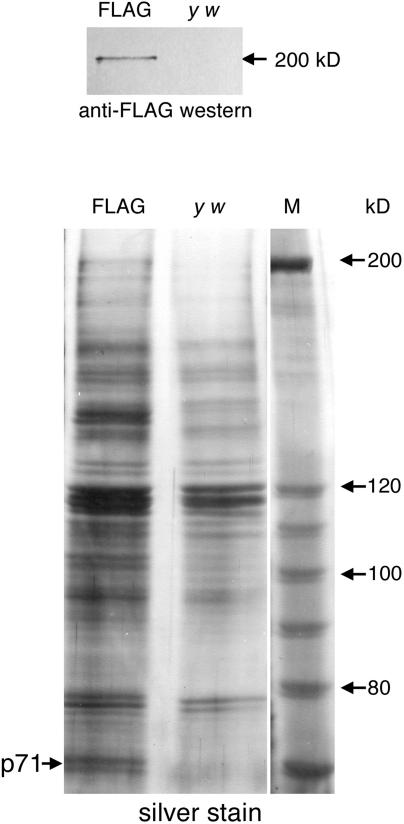
A 71-kDa Protein Co-Purifies with FLAG-Nipped-B by Anti-FLAG Affinity Chromatography Nuclear extracts of *y w;* P[ *Chip-FLAG-Nipped-B, w*+] (FLAG) embryos and *y w* control embryos were bound to anti-FLAG beads, washed, and eluted with FLAG peptide as described in the text. FLAG-Nipped-B fusion protein was detected in the *y w;* P[ *Chip-FLAG-Nipped-B, w*+] eluate, but not in the *y w* eluate, by anti-FLAG Western blot (top panel). Other eluted proteins were detected by silver stain (bottom panel). A 71-kDa protein specific to the FLAG-Nipped-B eluate was identified by mass spectrometry as the product of the *Drosophila CG4203* gene. It is closely related to the human MAU-2 protein. Higher molecular weight bands specific to the FLAG-Nipped-B extract contain multiple proteins whose identities could not be established unambiguously. In the bottom panel, the p71 protein and several other proteins, including the markers, became doublets when the gel was dried for photography. All appeared as single bands before drying.

To identify candidate protein partners for Nipped-B, FLAG-tagged Nipped-B was expressed from a transgene in *D. melanogaster.* Beads coated with anti-FLAG antibodies were used to pull out FLAG-Nipped-B from embryo extracts (see Materials and Methods). Multiple proteins bound to the anti-FLAG beads with the FLAG-Nipped-B extract, but not with the control, an extract of *y w* embryos. A 71-kDa protein found to be specific to the FLAG-Nipped-B extract in all experiments ( Figure 1, bottom panel) was excised from a silver-stained gel and processed for mass spectrometry (see Materials and Methods). After querying protein databases with selected mass values, the 71-kDa protein was identified as the product of the *Drosophila CG4203* gene. We were unable to identify other bands specific to the FLAG-Nipped-B extract because they contained multiple proteins. Following these studies, a global yeast two-hybrid screen in D. melanogaster provided confirmatory support for our findings. In this study, Nipped-B was reported to interact with several proteins, but in the reciprocal screen several of these did not appear to interact with Nipped-B; one that did, the CG4203 protein, was found to bind to Nipped-B only [ 38].

In initially independent projects we also sought protein partners for delangin, the human homolog of Nipped-B and yeast Scc-2. Screens with baits from the highly conserved C-terminal region of delangin (see Materials and Methods) identified a variety of possible protein partners but did not identify the human homolog of the CG4203 protein, KIAA0892 (unpublished data). However, reciprocal screening using a KIAA0892 bait to screen HeLa and skeletal muscle yeast two-hybrid libraries (see Materials and Methods) identified a single protein partner, delangin, and the region containing the KIAA0892-binding sequence was found to be restricted to the poorly conserved N-terminal region (unpublished data).

**Figure 2:**
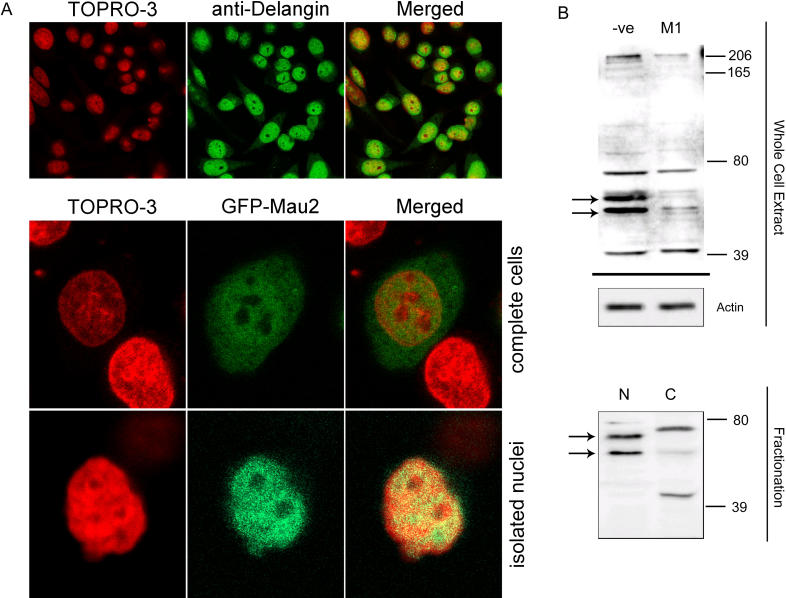
Human MAU-2 and Delangin Are Nuclear Proteins (A) Confocal microscopy studies on HeLa cells. The nuclear localization of delangin is illustrated in the top panels using a FITC-labeled secondary antibody detecting monoclonal rat anti-human delangin (see Materials and Methods). Panel on the right shows merging of DNA image (left panel, red) and delangin staining (center panel, green). Expression of a GFP-human MAU-2 fusion protein revealed by confocal fluorescence microscopy of transiently transfected HeLa cells is shown in the center and bottom panels. The fusion protein appears present in both the nucleus and the cytoplasm, sometimes with a strong nuclear localization, as shown in the center panels, but in other cells cytoplasmic expression predominated. Bottom panels show retention of the GFP-human MAU-2 protein in nuclei isolated from transiently transfected HeLa cells and subsequently extracted in 0.5% Triton-X. Center and bottom panels show DNA staining with TOPRO3 (left), the GFP fluorescence signal (center), and merged images (right). (B) Nuclear location for epitopes specific to human MAU-2. Top panel: Antisera against human MAU-2 cross reacted with four major bands in whole cell extracts from HeLa cells that had been treated with the negative (-ve) control siRNA oligonucleotide. The two bands indicated by the arrows were severely reduced in intensity (by ˜90%) when the same antisera was used to blot whole cell extracts from HeLa cells that had been subjected to human MAU-2 siRNA, using the M1 siRNA oligonucleotide (see Materials and Methods). Beta actin is shown as loading control. Bottom panel: HeLa cells were separated into cytoplasmic (C) and nuclear (N) fractions (see Materials and Methods). When these fractions were immunoblotted with human MAU-2 antisera, the bands specific to human MAU-2 were almost exclusively detected in the nuclear fraction, while the background bands appeared to be cytoplasmic.

**Figure 3:**
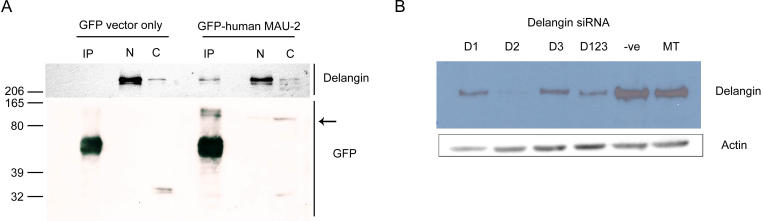
Co-Immunoprecipitation Studies Support Interaction between Delangin and Human MAU-2 (A) Delangin co-immunoprecipitates with a GFP-human MAU-2 fusion protein. HeLa cells were transiently transfected with a GFP-human MAU-2 fusion protein construct (see Materials and Methods) or, as a negative control, with the GFP vector on its own. Cells were fractionated into nuclear (N) and cytoplasmic (C) fractions, and aliquots of the nuclear fractions were used for immunoprecipitation with an anti-GFP antibody to generate immunoprecipitation fractions. Individual fractions were size fractionated by SDS-PAGE and immunoblotted, using antibodies against delangin (top panel) or against GFP (bottom panel) as described in Materials and Methods. The immunoprecipitation fraction from immunoprecipitating the GFP-human MAU-2 fusion protein reveals a specific band at ˜100 kDa (arrow) that is not found in the negative control sample. This is the size expected for the fusion protein (˜70 kDa for human MAU-2 and ˜ 30 kDa for GFP). The upper panel shows that the delangin antibodies identify a band of ˜ 300 kDa that is co-precipitated in the GFP-human MAU-2 immunoprecipitation sample but not in the GFP vector-only control sample. (B) Delangin siRNA. In order to validate the specificity of the monoclonal antibody to delangin, HeLa cells were transfected with siRNA oligonucleotides designed to knock down delangin using individual oligonucleotides D1–D3 (see Materials and Methods) or all three combined, D123. D2 produced the most effective delangin knockdown. The same band that was recognized by the antibody in the immunoprecipitation fraction (see A) was knocked down by ˜ 90% in cells transfected with the D2 siRNA oligonucleotide, when referenced against negative (−ve) and mock-transfected HeLa cell (MT) controls.

It has recently become clear that the previously uncharacterized *CG4203* and *KIAA0892* genes are orthologs of *C. elegans mau-2,* a gene involved in guiding the migration of cells and axons during development [ 37]. For the sake of simplicity, we will henceforth refer to the protein products of *CG4203* and *KIAA0892* as *Drosophila* MAU-2 and human MAU-2 respectively. Nipped-B and vertebrate delangins are predicted to have multiple nuclear localization sequences (see Materials and Methods), and in accordance with bioinformatics predictions both Nipped-B [ 24] and delangin ( Figure 2A, top panels) are nuclear proteins. Bioinformatic analysis of the C. elegans MAU-2 protein sequence and its human and D. melanogster orthologs fails to identify any such sequences ([ 37] and unpublished data), and a green fluorescent protein (GFP)-MAU-2 fusion protein was previously found to be located in the cytoplasm [ 37]. Following transient transfection of HeLa cells in the present study, GFP-human MAU-2 fusion protein constructs showed expression in both nucleus and cytoplasm. In some cells the proportion of nuclear expression was comparatively high ( Figure 2A, center panels), but often cytoplasmic expression predominated (see Figure 3A), possibly as a consequence of artificial over-expression. Nuclei that had been isolated from the transfected cells and extracted in Triton X-100 continued to show a high content of the GFP fusion protein ( Figure 2A, bottom panel), suggesting that this protein could be bound to chromatin. Polyclonal antisera raised against a GST-human MAU-2 fusion protein (see Materials and Methods) appeared to cross-react with other proteins in addition to MAU-2 ( Figure 2B, upper panel), making them ill-suited to immunofluorescence studies. Nevertheless, the specificity could be verified by short interfering RNA (siRNA) knockdown of human MAU-2. As seen in Figure 2B, upper panel, the two prominent bands in the expected size range (shown by arrows) appeared to be specific to MAU-2 and could represent alternative isoforms or be attributable to post-translational differences. Upon separation into nuclear and cytoplasmic fractions, the bands could be seen to be almost exclusively associated with the nuclear fraction ( Figure 2B, lower panel), suggesting that human MAU-2, like delangin, is a nuclear protein.

To validate the delangin-human MAU-2 interaction, we carried out co-immunoprecipitation studies ( Figure 3A). We transfected HeLa cells with a GFP-human MAU-2 fusion protein construct, prepared nuclear and cytoplasmic fractions, and carried out co-immunoprecipitation with anti-GFP antibodies on aliquots of the nuclear extract (see Materials and Methods). Consistent with the microscopy studies above, the anti-delangin antibody signal was largely nuclear. Immunoblotting of immunoprecipitated fractions with an anti-GFP antibody revealed a band that is specific to the immunoprecipitate with the GFP-human MAU-2 fusion protein and of the expected size ( Figure 3A, lower panel). A delangin-specific monoclonal antibody, whose specificity had been validated by siRNA knockdown experiments (see below and Figure 3B), showed that a band close to the expected size for delangin co-precipitates with the GFP-human MAU-2 fusion protein but not with a GFP vector control ( Figure 3A, upper panel).

### Members of the Metazoan MAU-2 Family Are Evolutionarily Related to S. cerevisiae Scc4

The wider (evolutionary) significance of Scc4 has been difficult to gauge. Whereas orthologs of Scc2 can readily be detected in apparently any eukaryote where there is sufficient sequence information, Scc4 appears at first sight to have been comparatively poorly conserved during evolution [ 9]. Searching contemporary sequence databases using standard BLASTP or tBLASTN with a Scc4 protein query identifies significant matching to sequences from a very limited number of organisms, all of them members of the hemiascomycetes group of fungi. They include various *Saccharomyces* and *Kluymoveres* species, Candida glabrata and *Ashbya gossypii,* but no homologs can be identified in this way in other fungal groups, such as in *Neurospora* species or *S. pombe,* or in any animal or plant genome (unpublished data). The comparable sizes of Scc4 (624 amino acids) and members of the metazoan MAU-2 family (593–632 amino acids) suggested that they could nevertheless be related. To identify metazoan homologs of Scc4 we used PSI-BLAST ([ 39], see Materials and Methods). The search converged after five rounds identifying 11 homologous proteins from various yeast species. The highest scoring human protein was KIAA0892, the human MAU-2 ortholog, with a score of 36 bits, corresponding to a non-significant E-value of 2.6.

**Figure 4:**
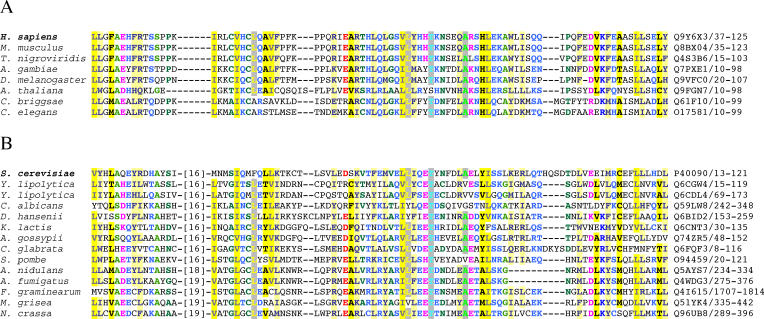
Alignment of the Conserved N-terminal Region of the Scc4/MAU-2 Family (A) Alignment of animal and plant MAU-2 homologs referenced against the human MAU-2 sequence shown at the top. (B) Alignment of fungal Scc4 homologs referenced against the S. cerevisiae Scc4 sequence shown at the top. Numbers in parentheses at left refer to a short sequence of 16–19 amino acids that has been omitted for clarity. Protein database accession numbers at right are followed by amino acid co-ordinates for the N-terminal sequences that have been aligned. The alignment was made with the MAFFT alignment tool [ 62] and colored using the CHROMA software to highlight conserved residues [ 63].

Although one would expect matches with the above score by chance, the match was in the N-terminal region of both Scc4 and human MAU-2, which is the most conserved region in the yeast homologs. To validate the above finding, we started with the human MAU-2 sequence and carried out a reciprocal PSI-BLAST search to see if we could identify Scc4. In the first round, vertebrate, invertebrate, and plant homologs of MAU-2 were identified. By round three a number of the yeast homologs of Scc4, identified above, were found confirming the relationship of Scc4 with the metazoan MAU-2 family. The alignments of the homologs identified show that the N-terminal sequences of the metazoan MAU-2 family members are the sequences most evolutionarily related to Scc4 ( Figure 4) and are likely to be of evolutionarily ancient functional significance. In round four a large number of tetratricopeptide repeat proteins were identified, suggesting that Scc4 family members are composed of tetratricopeptide repeats, structural bi-helical repeats implicated in protein–protein interactions [ 40].

### Delangin and Human MAU-2 Interact through N-Terminal Binding Sequences

**Figure 5:**
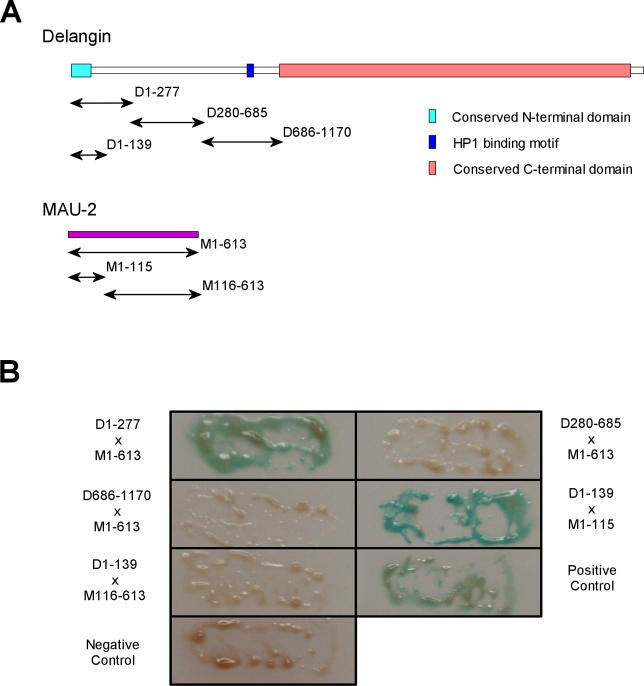
Physical Interaction between Delangin and Human MAU-2 Involves N-Terminal Binding Sites (A) Structure of delangin and human MAU-2 proteins and the positions of test constructs referred to in (B). (B) A yeast two-hybrid-based system was employed to test interaction between components of the delangin and human MAU-2 proteins as illustrated in (A) (see Materials and Methods). The system incorporated a β-galactosidase colony-lift filter assay so that positive interactions were scored by a blue color; colorless colonies signified no interaction. Full-length human MAU-2 was tested with different N-terminal components of delangin, spanning amino acids 1–277, amino acids 280–685, or amino acids 686–1170. Further mapping localized the N-terminal binding site to amino acids 1–139 of the delangin protein, which showed very strong interaction with an N-terminal human MAU-2 fragment spanning amino acids 1–115, but no binding to the remainder of the MAU-2 sequence from amino acids 116–613. A positive control showed interaction between murine p53 and SV40 large T-antigen expressed from constructs cloned in the vectors pGBK-T7 and pACT-2, respectively. A negative control demonstrated lack of interaction between the same vectors alone.

A reciprocal yeast two-hybrid library screen with a full-length human MAU-2 bait indicated that the binding site on delangin mapped to the N-terminal region upstream of amino acid 1171 (unpublished data). We subsequently tested possible yeast two-hybrid interactions between different N-terminal delangin components and the human MAU-2 sequence. As shown in Figure 5, individual constructs containing amino acids 280–685, or 686-1170 of delangin, did not recognize the human MAU-2 sequence, but one did that contained amino acids 1–277.

The latter fragment includes a sequence of approximately 100 amino acids at the extreme N-terminal end that is a distinctive component of the N-terminal region. Whereas the extended N-terminal region is generally poorly conserved and does not possess recognizable secondary structure motifs (see Materials and Methods), the first 100 or so amino acids are comparatively strongly conserved and are predicted to have a high helical content. To investigate this region we tested a shorter fragment including this sequence, and we simultaneously tested the possibility that the delangin binding site on human MAU-2 might be located close to the N-terminus, given the comparatively high conservation of this region within the wider Scc4/MAU-2 family ( Figure 4). The data obtained suggest that the delangin binding site on human MAU-2 is indeed confined to the N-terminal region (amino acids 1–115), and that the region upstream of amino acid 139 on the delangin protein carries the most important determinants for binding human MAU-2 ( Figure 5).

### siRNA-Mediated Gene Knockdown in HeLa Cells Reveals That Human MAU-2 Is Functionally Related to Scc4

**Figure 6:**
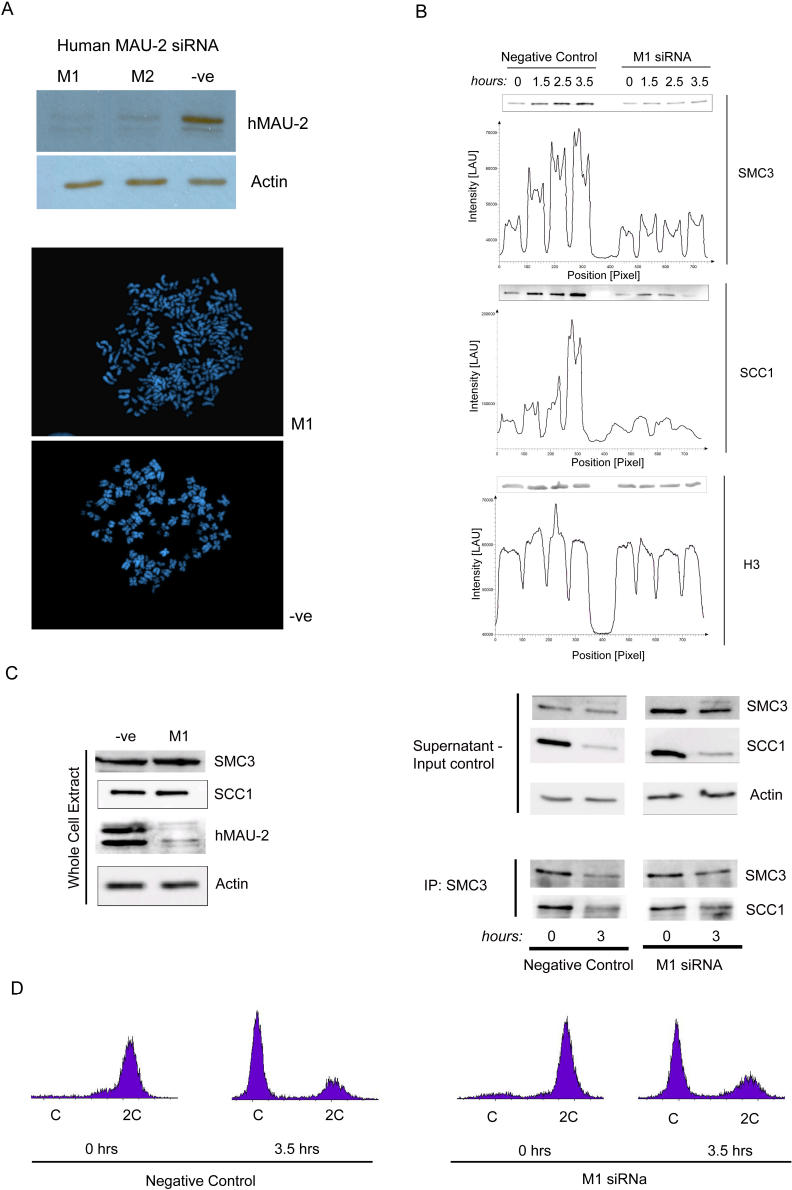
Human MAU-2 Regulates Sister Chromatid Cohesion and Is Required for Loading Cohesins onto Chromatin (A) Assay for PSCS. HeLa cells were transfected with siRNA oligonucleotides (M1 and M2) designed to knock down human MAU-2 (hMAU-2). The cells were synchronized at the G2/M stage by addition of nocodazole. After 3 h, the cells in the supernatant were collected and knockdown efficiency was assayed by immunoblotting using specific antibodies against human MAU-2. Top panel, both M1 and M2 effectively knocked down human MAU-2 (˜90% and 80% knockdown, respectively) when referenced against a negative (-ve) control oligonucleotide supplied by the manufacturer; beta actin is shown as a loading control. Metaphase spreads were prepared (see Materials and Methods) from these synchronized cells and assayed for PSCS separation. Middle panel, an example of PSCS in a metaphase of HeLa cells transfected with M1. Bottom panel, a metaphase from HeLa cells transfected with a negative (-ve) control oligonucleotide (see Materials and Methods). (B) By cohesin loading assay. HeLa cells transfected with a negative control oligonucleotide or subjected to human MAU-2 knockdown using the M1 oligonucleotide were synchronised in G2/M by nocodazole treatment and then released to progress into the cell cycle. Chromatin fractions prepared from aliquots collected at 0, 1.5, 2.5, and 3.5 h were subjected to Western blotting with anti-SMC3 and anti-SCC1 antibodies to monitor loading of cohesin on the chromatin. Histone H3 was used as a loading control (lower panel). The intensity of the bands was quantified as described in Materials and Methods. (C) Left panels: Western blotting of whole Hela cell extracts that had been subjected to M1 siRNA knockdown or controls shows that the effects seen in (B) did not result from non-specific knockdown of cohesins (SMC3 and SCC1). Panels to the right: to assess the integrity of the cohesin complexes present in the supernatant, SMC3 was immunoprecipitated (see Materials and Methods) and co-immunoprecipitation of SCC1 was tested. The ratio of SMC3:SCC1 present in the immunoprecipitated samples is identical in negative control cells and cells subjected to human MAU-2 knockdown indicating that the integrity of the cohesin complexes is not affected. In the negative control there is a marked difference in the amount of immunoprecipitated SMC3 between time 0 and 3 h later, as in these cells cohesin gets loaded on the chromatin and there is fewer SMC3 available for immunoprecipitation in the supernatant (see B). In contrast, in cells subjected to human MAU-2 this difference is notably smaller, as in these cells cohesin fails to load on the chromatin to a comparable extent (see B). (D) Cohesin-loading defects observed in cells subjected to human MAU-2 knockdown are not the result of cell-cycle arrest. HeLa cells were treated as in (B) and aliquots were collected at 0 and 3.5 h. The cell-cycle profile of the cell population in each sample was analyzed by flow cytometry.

The evolutionary relationship of metazoan orthologs of MAU-2 to budding yeast Scc4 suggested the possibility of a conserved role in sister chromatid cohesion. To examine this, we used two specific siRNA oligonucleotides, M1 and M2 (see Materials and Methods) to knock down human MAU-2 in HeLa cells and prepared metaphase spreads to assess any possible effect on sister chromatid cohesion. As shown in the top panel of Figure 6A, both M1 and M2 produced an effective knockdown, but the effect was significantly greater in the case of M1. Metaphase chromosome preparations from HeLa cells, subject to knockdown with M1, reproducibly showed a very significantly increased frequency of PSCS when referenced against controls, with some metaphases showing extensive PSCS that was never seen in controls ( Figure 6A, bottom panel). Out of 300 metaphases from HeLa cells subjected to M1 siRNA knockdown, we found a total of 547 dissociated chromosome pairs (3.96%), compared to 23 (0.16%) in an equivalent number of controls ( *p* < 10 ^−6^).

We followed up by asking whether, like Scc4, human MAU-2 is also involved in loading cohesins onto chromatin. HeLa cells subjected to human MAU-2 knockdown were synchronized in G2/M by nocodazole treatment and then released to progress into the cell cycle. Aliquots taken at regular intervals from cells released from the nocodazole block were fractionated to generate chromatin and supernatant fractions (see Materials and Methods) that were then tested for the presence of the cohesin subunits SMC3 and SCC1. As can be seen in Figure 6B, following the release from the nocodazole block, cohesin subunits start to be very quickly loaded on the chromatin in the control cells ( Figure 6B, upper and middle panel, left) but fail to do so in cells in which human MAU-2 had been knocked down using the M1 oligonucleotide ( Figure 6B, upper and middle panels, right). The cohesins (SMC3 and SCC1) in the supernatant could be readily co-immunoprecipitated suggesting that the complexes still form normally after knockdown of human MAU-2 ( Figure 6C, right panels). In the samples from control cells there is a difference between the amounts of cohesins immunoprecipitated at times 0 h and 3 h, respectively, but this difference is less obvious in the samples from cells in which human MAU-2 has been knocked down ( Figure 6C, bottom right panels). In accordance with the results in Figure 6B, cohesins are loaded on the chromatin in the control cells leaving less of them to be immunoprecipitated from the supernatant. Conversely, cohesins accumulate in the supernatant from the knocked down cells; therefore, the difference is much smaller between the amounts of cohesins immunoprecipitated at the two time points ( Figure 6C, bottom right panels). The above effects did not result from non-specific knockdown of the cohesins ( Figure 6C, left panels) or failure of the cells to progress through the cell cycle following siRNA knockdown ( Figure 6D).

### MAU-2 and PQN-85 Regulate Chromosome Segregation in Early C. elegans Embryos

To assess whether MAU-2 and PQN-85 (the C. elegans Scc2 ortholog) have chromosomal functions in addition to their developmental roles, we carried out RNA interference (RNAi) experiments in C. elegans to knock down *mau-2* and *pqn-85* expression. To visualize RNAi-induced abnormalities in chromosome segregation we used a histone::GFP strain of C. elegans (see Materials and Methods). As a control we also knocked down a cohesin subunit. To avoid potential problems with multiple paralogs (which exist in the case of SCC-1 [ 41]), we targeted *scc-3,* a single copy gene that is known to be essential for sister chromatid cohesion in C. elegans [ 42].

**Figure 7:**
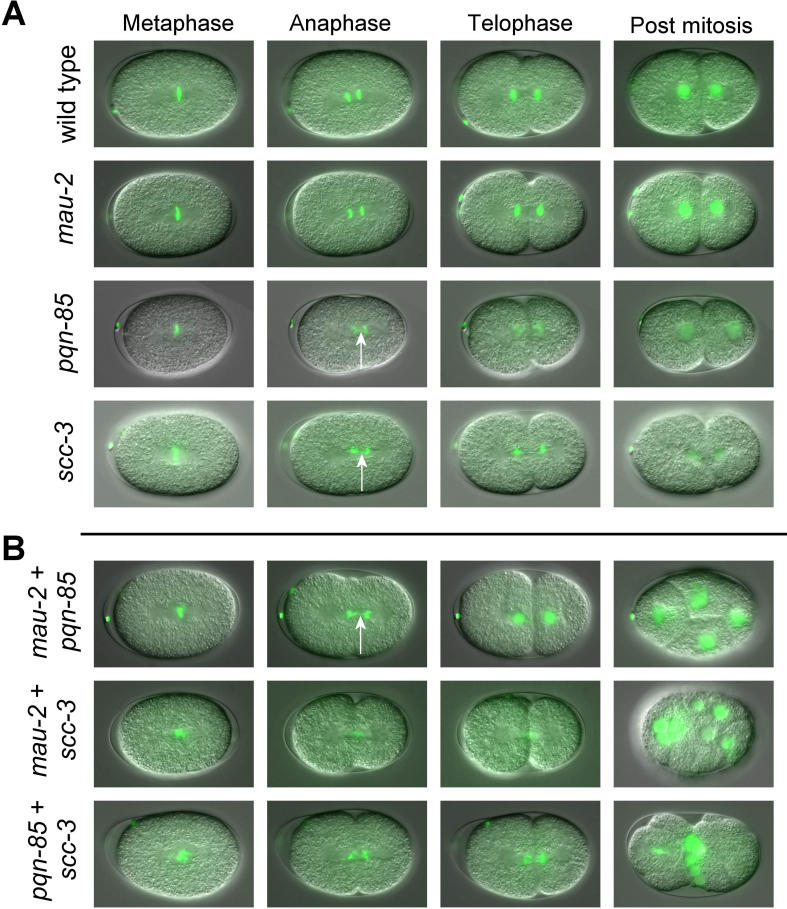
MAU-2 and PQN-85 Regulate Chromosome Segregation in Early C. elegans Embryos (A) Individual RNAi knockdowns. Chromosome-segregation defects are not obvious in the progeny of *histone::GFP* hermaphrodites injected with double-stranded *mau-2* RNA. However, lagging anaphase chromosomes were evident in the case of *pqn-85*(RNAi) and *scc-3*(RNAi) embryos (white arrows). (B) Double RNAi knockdowns. Early *mau-2* + *pqn-85* (RNAi) embryos showed chromosome lagging (white arrow) where some ensuing cells appear to be unaffected and others have multiple and misshapen nuclei (right image). Early *mau-2* + *scc-3* (RNAi) and *pqn-85* + *scc-3* (RNAi) embryos consistently showed severe chromosome segregation defects where the DNA does not appear to move to either pole. This results in the phenotype in which all cells either have multiple nuclei or have none at all. Precise assignment of cell-cycle stages was not possible because of the severity of the chromosomal phenotype.

Knockdown of *pqn-85* or of *scc-3* resulted in embryonic lethality with 100% penetrance, but no lethality was observed in the case of the wild-type embryos and embryos subjected to *mau-2* knockdown. Simultaneous knockdown of *mau-2* plus *pqn-85,* of *mau-2* plus *scc-3,* or of *pqn-85* plus *scc-3* all resulted in embryonic lethality with 100% penetrance (unpublished data). The embryos subjected to individual *pqn-85* or *scc-3* knockdown showed lagging chromosomes at anaphase, but equivalent knockdown of *mau-2* did not result in obvious abnormalities of chromosome segregation ( Figure 7A). However, simultaneous knockdown of *mau-2* plus *scc-3* or of *pqn-85* plus *scc-3* reproducibly produced much more severe phenotypes than those from any of the single knockdowns. Both of these double knockdowns consistently resulted in a catastrophic failure of chromosome segregation. The resulting phenotypes were identical, with 100% of ensuing cells having either multiple and misshapen nuclei or no nuclei (22 embryos were scored in the case of the *mau-2* plus *scc-3* knockdown, and 27 were scored in the case of the *mau-2* plus *scc-3* knockdown; Figure 7B).

Of the embryos that had been subjected to simultaneous knockdown of *mau-2* plus *pqn-85*, chromosomal defects were evident in 48% of cells of the 17 embryos scored. By comparison, embryos that had been subjected to single *scc-3* knockdown showed chromosomal defects in 58% of cells of 15 embryos scored, while embryos subjected to single *pqn-85* knockdown showed defects in 28% of the cells in 11 embryos. The data show increased severity of chromosomal phenotypes for embryos subjected to simultaneous knockdown of *mau-2* plus *scc-3* or of simultaneous knockdown of *mau-2* plus *pqn-85,* when referenced against the respective phenotypes for the single knockdowns and are consistent with a role for *mau-2* in chromosome segregation. The comparatively reduced severity of the *mau-2* plus *pqn-85* chromosomal phenotype when referenced against that for the *scc-3* knockdown, is consistent with the idea that MAU-2 and PQN-85 form part of a single functioning complex that also acts with cohesin (SCC-3) in regulating chromosome segregation.

### Targeted Gene Knockdown in X. tropicalis Embryos Reveals That MAU-2 Operates in Similar Developmental Pathways as Delangin

**Figure 8:**
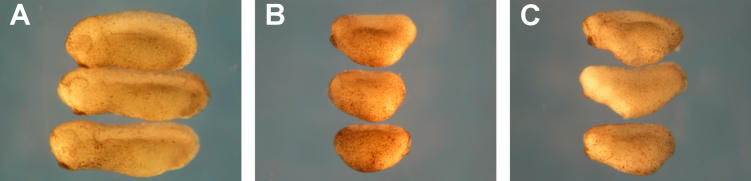
Delangin and MAU-2 Regulate Similar Processes in Xenopus tropicalis Embryonic Development Antisense morpholino oligonucleotides (MO) were used to target specific mRNAs to inhibit production of X. tropicalis delangin or MAU-2 (see Materials and Methods). Embryos injected with control MO exhibit normal development (A). Embryos targeted to knock down delangin or MAU-2 both exhibit a delay in development from gastrula stages relative to control MO-injected embryos, however they look normal at this stage. By late tailbud stage (stage 28), delangin morphants (B) are severely truncated along the A-P axis and ventralized, exhibiting retarded dorsal tissue development, particularly in the neural tube and somites. Head, eye, and tail development are also defective. MAU-2 morphants (C) exhibit a very similar but less severe phenotype than is evident in delangin morphants, including shortening of the A-P axis, ventralization and defects in neural, somite, head, eye, and tail development relative to the control MO-injected embryos.

The possibility of related developmental roles for metazoan Scc2 and Scc4 homologs was also suggested by some similarities of mutant phenotypes for the C. elegans Scc2 ortholog, *pqn85* (J. Ahringer, unpublished data, detailed in [ 29]), and *mau-2*. To investigate a role in regulating vertebrate development, we designed specific morpholino antisense oligonucleotides to knock down X. tropicalis MAU-2 in one-cell embryos (see Materials and Methods). Knockdown of X. tropicalis MAU-2 or delangin at the one-cell stage resulted in similar abnormalities of development. In each case there was a marked delay in development from gastrula stages when referenced against embryos that had been treated with control morpholino oligonucleotides (see Materials and Methods). By stage 28 (late tailbud stage), delangin morphants could be seen to be severely truncated along the anterior-posterior axis and ventralized, exhibiting retarded dorsal tissue development, particularly in the neural tube and somites (compare Figure 8A to Figure 8B). Head, eye, and tail development were also defective. The phenotype of the MAU-2 morphants, albeit less severe than that of the delangin morphants, shows many similarities, including shortening of the A-P axis and ventralization ( Figure 8C), and defects in neural, somite, head, eye, and tail development were also observed relative to embryos that had been injected with control morpholino oligonucleotides.

## Discussion

### Evolutionary Conservation of the Scc2 and Scc4 Protein Families

The precise roles of metazoan Scc2 and Scc4 homologs remain to be elucidated. Cross-species comparisons for the two protein families are instructive. First, there is the apparently striking difference in evolutionary conservation. Strong conservative selection of much of the protein sequence means that Scc2 can readily be detected in apparently all eukaryotes where there is adequate sequence information. In contrast, standard BLASTP and tBLASTN searches can identify Scc4 homologs only in certain fungal species that are closely related to *S. cerevisiae.* Nevertheless, the PSI-BLAST analyses have shown that Scc4 has more distantly related homologs, including a previously unstudied S. pombe homolog.

Part of the difference in evolutionary conservation of these two protein families could relate to differential requirements for protein interaction. Multiple candidate protein partners are revealed following screens for protein partners using Nipped-B [ 38] or delangin (our unpublished data), and it may be that the conserved C-terminal domain is under strong selection pressure to recognize various proteins and/or play a vital role in cohesin binding [ 12]. In contrast, reciprocal screens using CG4203, the D. melanogaster MAU-2 ortholog [ 38], or human MAU-2 (present study), have revealed a single protein partner: Nipped-B or delangin, respectively. If Scc4 does not directly bind cohesins, perhaps early in eukaryote evolution the only selection pressure on Scc4 was to recognize and bind Scc2. The resulting comparative lack of selection pressure could have allowed very considerable species divergence in Scc4 sequence. However, the marked sequence conservation of Scc4 homologues in some metazoan lineages may reflect subsequent acquisition of additional essential functions (see below).

### Evolution of Function in the Scc2 Protein Family

Fungal Scc2 family members have been well studied and have been shown to regulate various aspects of chromosome function, including sister chromatid cohesion, chromosome segregation, and double-strand DNA repair (see Introduction and summary table in [ 29]). Recently, metazoan homologs have also been implicated in sister chromatid cohesion (see Introduction), but initial investigations focused on their roles in early development. *Nipped-B* was isolated in a screen to identify factors that regulate long-range activation of *Drosophila cut;* the *cut* gene is activated by a remote enhancer located 85 kb upstream, and this activation is regulated by *Nipped-B* [ 26]. However, unlike for other *cut* activators, the effect of *Nipped-B* is much more significant when *cut* expression is limited by insertion of a *gypsy* transposon between *cut* and its enhancer, than when components of the enhancer are deleted. The effect of the inserted *gypsy* transposon is to act as an insulator, and because mutations in *Nipped-B* have their greatest effect when *cut* activation is compromised by *gypsy* insulator action, the normal Nipped-B protein has been envisaged to facilitate interaction between the remote enhancer and promoter of *cut.* A similar situation applies to activation of another *Nipped-B* target gene, *Ultrabithorax* [ 26]. Other target genes remain to be identified, but genetic studies show that some *Nipped-B* mutations suppress certain *Notch* mutant phenotypes [ 26]. Delangin was identified by investigating the molecular basis of a developmental malformation, Cornelia de Lange syndrome (see [ 29]), and can be expected to be functionally similar to Nipped-B.

While roles in basic chromosomal function have been conserved in the Scc2 protein family, a key question concerns the extent to which the roles in gene regulation—that are apparent for Nipped-B and expected for vertebrate delangins—are metazoan novelties. Gene regulation in metazoan cells, unlike in simple eukaryotic cells such as the budding yeast, often involves interaction between promoters and *cis*-acting transcriptional activators and repressors that may be separated by long distances, often tens or hundreds of kb. In addition to architectural factors like HMG proteins that facilitate such interactions over short distances (hundreds of base pairs), other factors are required to support interactions over long distances in metazoan cells, and the screen that identified *Nipped-B* was carried out to identify factors of this type. It is possible, therefore, that during metazoan evolution Scc2-type proteins became much more important in gene regulation while continuing to fulfill ancestral functions in chromosomal regulation. However, there is a close relationship between sister chromatid cohesion and transcriptional silencing in unicellular yeasts (see [ 43] and references therein), and Scc2-type proteins in simple eukaryotes could also be involved in gene regulation.

### Evolution of Function in the Scc4 Protein Family

While overall sequence divergence between Scc4 family members has generally been much more marked than between Scc2 family members, the role in sister chromatid cohesion and cohesin loading onto chromatin seems to have been conserved from budding yeast to humans. Consistent with this, the RNAi studies in C. elegans show that *mau-2* interacts with *pqn-85* and with *scc-3* in regulating chromosome segregation. Further evidence comes from fission yeast where the product of the *SPAC1687.18c* gene, which we have identified as a highly diverged *Scc4* homolog (see Figure 4), has recently been found to bind to Mis4, the S. pombe Scc2 ortholog, and to function in cohesin loading (J. P. Javerzat, personal communication).

Although from a broad metazoan perspective, MAU-2 sequence conservation does not at first appear to be so high, the degree of sequence conservation is very pronounced in more complex metazoans and vertebrates. The sequence identity between essentially full-length human and C. elegans MAU-2 is only 21%, somewhat less than the 37% identity between the conserved C-terminal domains of delangin and its C. elegans ortholog PQN-85. However, almost full-length comparisons between human MAU-2 and more complex metazoan orthologs give high sequence identities as follows: D. melanogaster, 49%; sea urchin, *Strongylocentrotus purpuratus,* 67%; *X. tropicalis,* 96%; and chick, 97% (see the Supporting Information section below for sequence accession numbers and comments).

The latter figures are much higher than those obtained in equivalent ortholog comparisons for even the highly conserved C-terminal domain of delangin (unpublished data). The data are consistent with increased selection pressure in complex metazoans to maintain sequence conservation across essentially the full-length sequence of Scc4 homologs. The participation of a C. elegans Scc4 homolog in axon and cell migration guidance was unexpected, and it will be important to test if other metazoan homologs have similar functions. Whatever the origin—whether acquisition of an additional essential function in metazoans or variation of an ancestral function—the very high sequence identities between the Scc4 homologs of complex metazoans may reflect the increasing importance of ensuring correct migration of axons and cells in complex metazoans.

From Figure 8, the X. tropicalis homolog appears to function in the early embryo in developmental pathways that are similar to those in which delangin participates. A large-scale morpholino antisense oligonucleotide knockdown approach has identified similar phenotypes for knockdown of mRNA specifying a variety of other proteins, including the 14-1-3–3 and Emsy genes (Rana et al., unpublished data). However, RNAi knockdown of *Drosophila* MAU-2 (see Materials and Methods) failed to elicit any obvious developmental phenotype, possibly as a result of ineffective knockdown, or possibly because of gene-dosage differences between species.

### Sister Chromatid Cohesion, Developmental Gene Regulation, and Pathogenesis

The dual nature of the role of metazoan Scc4 homologs in regulating both chromosomal functions and developmental processes mirrors that of the metazoan Scc2 homologs, and there appears to be functional co-operation between members of the two families in both chromosomal and developmental regulation. A variety of other genes that are directly involved in sister chromatid cohesion or chromosome condensation (or that are paralogs of such) are now also known to play important roles in gene/developmental regulation. Recent examples include the *ESCO2* gene that is involved in establishing sister chromatid cohesion and that is a locus for a developmental malformation, Roberts syndrome [ 44, 45], Scc1 homologs with distinct developmental functions in C. elegans [ 41], and clock gene paralogs that regulate chromosome cohesion in C. elegans [ 46] (see also other studies cited in [ 8]).

The data obtained in the present study also raised the question of whether human MAU-2 could, like delangin, be involved in pathogenesis. The precise roles of delangin and Nipped-B, and the connection between their involvement in sister chromatid cohesion and developmental gene regulation, remain to be elucidated. Mutations in *NIPBL* that cause CdLS are thought to be predominantly heterozygous loss of function mutations (see [ 29]). The ensuing reduced dosage of delangin could conceivably result in increased apoptosis during early development with loss of key tissue progenitor cells. Another possibility depends on whether, in addition to its general function in loading cohesins onto chromosomes, delangin could also regulate target genes by facilitating transient unloading of cohesins. Here, a failure to unload cohesins from regions that need to be transcribed could result in transcriptional defects in certain target genes that result in CdLS [ 31]. Consistent with this idea, a special *Drosophila pds5* mutation that decreases cohesin chromosomal binding increases *cut* gene expression during wing margin development [ 30].

The link between sister chromatid cohesion and regulation of cell and axon migration that has now been revealed by analysis of the metazoan MAU-2 family was unexpected. Conceivably, by affecting cell division, sister chromatid cohesion could affect the asymmetric segregation between daughter cells of determinants of cell fate. Altered cell fates of neurons and of cells (e.g., epidermal cells), which are the substrates on which axons migrate, could affect cell and axon migrations. Another possibility is that in addition to its role in chromosome segregation, MAU-2 also functions in a diferent, relatively independent, pathway that involves proteins directly linked to cell and axon migration.

Conservation of Scc2-Scc4 interaction in metazoans and phenotype similarity following knockdown of MAU-2 and delangin in X. tropicalis suggested the possibility that *KIAA0892* could be a CdLS gene. Studies using standard point mutation screening strategies have identified pathogenic *NIPBL* mutations in only 35%–50% of CdLS individuals tested [ 27, 28, 47– 50]. Whole *NIPBL* gene deletions are rarely seen (see [ 29]), and partial FISH-based screens have not suggested that medium-scale mutations spanning multiple exons are particularly common ([ 47] and unpublished data). However, after amplifying all exonic and neighboring intronic sequences from 18 classical CdLS individuals who had tested negative in *NIPBL* mutation screens (see Materials and Methods), we failed to find any evidence that *KIAA0892* is involved in the pathogenesis of CdLS (unpublished data). While the results cannot exclude *KIAA8092* as a minor CdLS gene, an alternative is that, because of the way it functions, the consequences of reducing its dosage may be less severe. Cutting Nipped-B or delangin levels to one-half of normal can result in profound phenotypes. However, recorded *mau-2* mutants are recessive, and it may be that a single defective *KIAA0892* allele may have little phenotypic consequences while two defective alleles are frequently lethal.

## Materials and Methods

### Antibodies.

Rat anti-delangin monoclonal antibodies (Absea Biotechnology, Beijing, China) were raised using C-terminal peptide immunogens NH2-CGTSGVRRRRSQRISQRIT and NH2-CGSWTEAKRRDGRKLV-OH. Rabbit anti-human MAU-2 polyclonal antibodies were obtained using a glutathione S-transferase (GST)-fusion protein containing amino acids 125 to 385 of human MAU-2. Rabbit polyclonal antibodies against GFP, human SMC3, and human SCC1 were purchased from Abcam (Cambridge, United Kingdom), as was a mouse monoclonal anti-beta actin antibody **.**

### Expression of FLAG-Nipped-B fusion protein in *Drosophila* and preparation of nuclear extract.

A FLAG-Nipped-B fusion cDNA was constructed from a full-length *Nipped-B* cDNA [ 26] and cloned downstream of a *Chip* gene promoter [ 51] in a pCaSpeR P-element vector [ 52]. An insertion of the resulting P-element vector that rescued viability of *Nipped-B* mutants was made homozygous and the resulting stock, which was wild-type for endogenous *Nipped-B,* was designated *y w;* P( *Chip-FLAG-Nipped-B, w+*) and used to make nuclear extract. Nuclear extract was prepared from 10- to 12-h-old *y w* and *y w;* P( *Chip-FLAG-Nipped-B, w+*) embryos as described elsewhere [ 53], except that the final extract was dialyzed against buffer (25 mM Hepes-KOH [pH 7.6], 90 mM KCl, 12.5 mM MgCl _2_, 0.1 mM EDTA, 20% (v/v) glycerol, 1 mM DTT, 0.5 mM PMSF, 1 mM benzamidine), clarified by centrifugation, and stored at −80 °C.

### FLAG-immunoaffinity purification and mass spectrometry analysis.

Nuclear extracts from embryos [250 mg each of *y w* or *y w;* P( *Chip-FLAG-Nipped-B, w+*)] were incubated overnight at 4 °C, using gentle agitation with anti-FLAG M2 affinity gel (Sigma, St. Louis, Missouri, United States). The resin was collected by centrifugation, washed with 10 volumes of 0.25-HEGN Buffer (25 mM Hepes-KOH [pH 7.6], 0.25 M KCl, 0.1 mM EDTA, 10% (v/v) glycerol, 0.1% (v/v) NP-40, 1 mM DTT, 0.1 mM PMSF). Bound proteins were eluted with 0.25-HEGN containing 0.5 mg/ml FLAG peptide (DYKDDDDK, Sigma). Fractions of the eluate were separated on 8% SDS-PAGE gels, and protein was visualized by silver stain. FLAG-Nipped-B was identified in the eluate by Western blot using an anti-FLAG M2 antibody (Kodak, 1:360 dilution), goat anti-mouse secondary (Jackson, 1:40,000 dilution) and the Protoblot AP System (Promega, Madison, Wisconsin, United States) for detection ( Figure 1, top panel). Gel-resolved protein was digested with trypsin, and the resulting peptides were batch-purified on a reversed-phase micro-tip [ 54]. Fractionated peptide pools were analyzed by matrix-assisted laser desorption/ionization reflectron time-of-flight (MALDI-reTOF) mass spectrometry (MS) (Ultraflex TOF/TOF, Bruker, Bremen, Germany) for peptide mass fingerprinting as described [ 55]. Selected pepitide ions (m/z) were taken to search the Celera-Berkeley *Drosophila* protein database using the PeptideSearch algorithm (Matthias Mann, Max-Planck Institute for Biochemistry, Martinsried, Germany; an updated version of this program is currently available as “PepSea” from Applied Biosystems/MDS Sciex, Foster City, California, United States). A molecular mass range up to twice the apparent molecular weight (as estimated from electrophoretic relative mobility) was covered, with a mass accuracy restriction of less than 35 ppm, and maximum one missed cleavage site allowed per peptide. To confirm PMF results with scores ≤ 40, mass spectrometric sequencing of selected peptides was done by MALDI-TOF/TOF (MS/MS) analysis on the same prepared samples, using the UltraFlex instrument in “LIFT” mode. Fragment ion spectra were taken to search NR using the MASCOT MS/MS Ion Search program [ 56], version 2.0.04 for Windows (Matrix Science, London, United Kingdom). Any tentative confirmation (Mascot score ≥ 30) of a PMF result thus obtained was verified by comparing the computer-generated fragment ion series of the predicted tryptic peptide with the experimental MS/MS data.

### Yeast two-hybrid-based library screening and validation of interaction between selected protein pairs.

Yeast two-hybrid library screening was performed essentially as specified by the library manufacturer (BD-Clontech, Palo Alto, California, United States) and as described previously [ 57]. Pre-transformed libraries from both HeLa cells and human skeletal muscle were screened with baits cloned into pGBK-T7. Multiple library representations were screened in both cases. Positive clones were identified following 10-d culture on quadruple dropout medium (minimal medium lacking adenine, leucine, tryptophan, and histidine), and plasmid rescue and sequencing of positive colonies was performed using standard techniques. Validation of potential interaction between selected regions of human delangin and human MAU-2 was achieved by cloning the relevant regions of the *NIPBL* and *KIAA0892* coding sequences into pGBK-T7, or pACT-2, transforming into yeast strains AH109 and Y187, mating the relevant haploid strains and selecting the diploid progeny as above. Positive and negative controls were provided by Clontech and are detailed in the legend to Figure 5.

### Identifying protein partners using strepatvidin pulldown of biotin-tagged constructs and mass spectrometry.

The adapted method of de Boer and colleagues [ 58, 59] was used to generate cDNA constructs specifying delangin fusion proteins with a 23-amino acid N-terminal tag that could be biotinylated in vivo (biotag) *.* A series of such constructs were designed that collectively specified overlapping fragments from the conserved C-terminal region of delangin (see text) and cloned into the expression vector, pEV-puromycin, which consists of the human β-globin locus control region sequences, the human β-globin promoter and the β-globin second intron. To identify proteins that interact with the C-terminus of delangin, C88 ^BirA^ mouse erytholeukemic cells (MEL) expressing the Escherichia coli BirA protein-biotin ligase [ 59], were stably transfected with pEV-biotag-delangin recombinants. Induction of MEL cells to differentiate upregulates β-globin. Since BirA and the biotag-delangin constructs are under the control of human β-globin LCR sequences, the C88 ^BirA/biotag-delangin^ cells also upregulate the biotin ligase and bio-tagged delangin fragments upon induction. 20 C88 ^BIR/biotag-delangin^ clones were isolated and induced to differentiate. Nuclear extracts were tested for presence of the fusion protein using a delangin-specific antibody and streptavidin horseradish peroxidase. Nuclear extracts prepared from induced different clones of cells were incubated with streptavidin-coated paramagnetic beads (Dynabeads M-280, Dynal, Oslo, Norway) and pulled down proteins separated by polyacrylamide gel electrophoresis. Proteins were trypsin digested, eluted, and analyzed by mass spectrometry (LC-MSMS).

### Cellular fractionation and intracellular protein localization.

HeLa cells were separated into nuclear and cytoplasmic fractions as described in [ 60]. Total RNA was extracted from HeLa cells using RNAwiz reagent (Ambion, Austin, Texas, United States). *KIAA0892* cDNA was obtained by RT-PCR using Invitrogen sSuperscript III reverse transcriptase (Invitrogen, Carlsbad, California, United States). The entire coding sequence was amplified using linkered primers ( TTGGATCCATGGCGGCTCAGGCGGCGGC and TTGTCGACCAGGAGGCTGGCCAGGCTGG) and cloned into pEGFP C1 (Clontech). All constructs were verified by sequencing. HeLa cells were plated at semi-confluence in Lab-Tek chamber flasks (Nunc, Roskilde, Denmark). Following fixation in cold methanol/acetone, the cells were blocked with serum-free protein block (Dako, Glostrup, Denmark) and incubated overnight in the relevant antibody. Following washing in PBS, a mixture of secondary antibodies (FITC-labeled donkey anti-rat and TRITC labeled donkey anti-rabbit, Jackson ImmunoResearch, West Grove, Pennsylvania, United States) was applied. Images were collected on a Zeiss LSM150 confocal microscope equipped with a META head and a Plan-APOCHROMAT 63x/1.4 lens using LSM software v4.3.

### GFP-human MAU-2 immunoprecipitation.

HeLa cells were transiently transfected (>70% transfection efficiency) with a GFP-human MAU-2 fusion protein construct or with the GFP vector only (pEGFP-C1, Clontech). After 20–24 h, the cells were examined under an inverted fluorescence microscope, washed in PBS, re-suspended in a hypotonic buffer containing 10 mM HEPES [pH 7.9], 10 mM KCl, 1.5 mM MgCl _2_, 0.34 M sucrose, 10% glycerol, 1mM DTT, Complete ^TM^ protease inhibitors cocktail (Roche, Basel, Switzerland), and 0.5 mM PMSF [ 59]. Cells were lysed by addition of Triton X-100 (to 0.2%) and incubated on ice for 10 min. Nuclei were collected by centrifugation at 1,300 × *g* for 5 min at 4 °C, washed once more in the same buffer, and collected as before. The nuclei were re-suspended in PBS containing 0.2% triton X-100 and protease inhibitors and lysed by passing them 20 times through a 25 G (5/8) syringe needle. The samples were pre-absorbed with sepharose-protein A. 0.5 μg of anti-GFP antibody was added to the nuclear lysate and the mixture was incubated overnight on a rotating wheel at 4 °C. 10-μl bed volume of sepharose-protein A beads was added to the tubes and incubated rotating for an additional 1 h at 4 °C. The beads were collected by centrifugation and washed 3 times in PBS supplemented with protease inhibitors. The beads were re-suspended and boiled in Laemmli sample buffer. Samples were separated by standard SDS-PAGE, transferred on PVDF membrane, and analyzed by immunoblotting with anti-GFP and anti-delangin antibodies (see above).

### Bioinformatics analyses.

Candidate nuclear localization signals were identified using the PSORTII program at http://psort.hgc.jp. Protein structure prediction used the PSIPRED server ( http://bioinf.cs.ucl.ac.uk/psipred) and the position-specific scoring matrix of Jones et al. [ 61]. Analyses with PSI-BLAST [ 39] involved using protein sequences to query the Uniprot database [version 6.1) with a default threshold E-value of 0.001. Sequence alignments were made with the MAFFT alignment tool [ 62] and colored using the CHROMA software to highlight conserved residues [ 63].

### RNAi in *D. melanogaster*

There are no mutations or deletions of the *CG4203* gene, so to test for in vivo functions of *CG4203,* we conducted RNAi, using a 790 bp *Xho*1- *Sal*1 fragment from the N-terminal half of the *CG4203* cDNA clone ( *Drosophila* Gene Collection, Open Biosystems, Huntsville, Alabama, United States) to make double-stranded RNA. The fragment was cloned into the *sym-puast* P-element vector [ 64] and then used to generate germline transformants [ 24]. This vector bi-directionally transcribes inserted DNA in response to the yeast Gal4 activator to make double-stranded RNA. We tested five independent insertions of the *CG4203* RNAi P-element with an *actin5c-gal4* ubiquitous driver, a *daughterless-Gal4* driver, and an *hsp70-Gal4* driver at 25 °C and 29 °C. We did not see a significant effect of *CG4203* RNAi on viability, or any obvious mutant phenotypes with any of the insertions under any of these conditions. In contrast, with the *act5c-gal4* and *daughterless*- *Gal4* drivers at 25 °C, and the *hsp70-gal4* driver at 29 °C, *Nipped-B* RNAi was lethal [ 24]. Similarly, RNAi knockdown of the rad21/mcd1/scc1 and stromalin/scc3 cohesin subunits was lethal with the *actin5c-Gal4* driver at 25 °C and semilethal with the *hsp70-Gal4* driver at 29 °C [ 24]. We also did not detect effects of *CG4203* RNAi on expression of the *cut* gene under conditions that *Nipped-B* RNAi decreased *cut* expression, and *rad21* and *stromalin/scc3* RNAi increased expression [ 24]. RNAi did not completely eliminate transcripts of any of the target genes even with the *act5c-Gal4* driver at 29 °C, and the Nipped-B RNAi lethal phase was later than that of null mutants, indicating that RNAi does not completely abolish expression [ 24]. Thus, we cannot rule out the possibility that we did not see effects on viability or *cut* expression with CG4203 RNAi because p71 is not as limiting as Nipped-B and cohesin.

### siRNA-mediated gene knockdowns.

Test siRNA oligonucleotides (Eurogentec, Invitrogen) included ones designed to knock down production of human MAU-2 (sense sequences from the *KIAA0892* gene are: UGUCUGAAUUGUACUGU CAAGAGAA [M1] and CGAGUGUGUAUAU ACGGGAAGGAAA [M2]) and delangin ( *NIPBL* sense sequences: U AACGAUACU GAAGAAGAA [D1], ACACU CAGAU GAAGAAGUA [D2], and GGACUCUAAU GCAGAAGAA [D3]). Negative control oligonucleotides were from Eurogentec. Transient transfection of HeLa cells used 200 nM oligonucleotides and Lipofectamine ^TM^ 2000 (Invitrogen) according to the manufacturer's instructions. After 24 h following transfection, the cells were washed in PBS, fresh medium was added, and a second round of siRNA transfection was carried out using the same protocol as for the first round. Knockdown efficiency was validated at 48 and 72 h after the second transfection by immunoblotting with human MAU-2- and delangin-specific antibodies (see above).

### Precocious sister chromatid separation assays.

HeLa cells were transfected with 200 nM of siRNA oligonucleotides. After 24 h cells were washed in PBS, fresh medium was added, and cells were transfected again with the siRNA oligos at the same concentration. 48 h after the second transfection, cells were washed in PBS and fresh medium containing 0.1 μg/ml nocodazole was added. After 3 h the cells in the supernatant were collected by centrifugation. Cells were re-suspended in 75 mM KCl and incubated at 37 °C for 5 min. Cells were collected by centrifugation and re-suspended in fix solution (3:1 methanol:acetic acid). The cells were re-centrifuged and re-suspended in fix solution again before dropping onto slides. The cells were then stained with DAPI, and a total of 300 metaphases were microscopically analyzed for PSCS. To provide an objective assessment, individual chromosome pairs were scored for evidence of PSCS.

### Cohesin loading assays.

HeLa cells subjected to MAU-2-specific siRNA knockdown, or to negative control siRNA, were synchronized in G2/M by nocodazole treatment (0.1 μg/ml as above) and then released to progress into the cell cycle. Chromatin fractions were prepared from aliquots collected at 0, 1.5, 2.5, and 3.5 h following release of the nocadazole block using a slight modification of the method of Méndez and Stillman [ 60], i.e., cells were trypsinized where needed and washed twice in ice-cold PBS, and this protocol was then followed exactly, except that cells were lysed in 0.2% Triton X-100. The output chromatin fractions were then further extracted in 0.6% Triton X-100 [3 mM EDTA, 0.2 mM EGTA, 1 mM DTT, protease inhibitors] and centrifuged at 13,000 rpm, 4 °C, through a 0.5 ml cushion of 1M sucrose. The supernatant was carefully removed, and the chromatin pellet was extracted in 0.5 M NaCl [3 mM EDTA, 0.2 mM EGTA, 1 mM DTT, protease inhibitors]. The chromatin was spun down through a sucrose cushion as above and boiled in Laemmli sample buffer. These samples were sonicated to break down the chromatin and then boiled for 5 additional min. Chromatin fractions were subjected to Western blotting with anti-SMC3 and anti-SCC1 antibodies to monitor loading of cohesin on the chromatin. Histone H3 was used as a loading control, and Western blot signals were quantitated by using a LAS-3000 luminescent image analyser (Fujifilm, Tokyo, Japan) and quantified using the AIDA software version 4.13.023 (Raytest). Immunoprecipitation of SMC3 was carried out from supernatant fractions resulting from the cellular fractionation described above. The supernatant fractions were diluted 1:4 in PBS supplemented with protease inhibitors and then pre-absorbed for 30 min at 4 °C with 30 μl of sepharose-protein A beads. 0.5 μg of anti-SMC3 antibody was added to the cleared samples that were then incubated overnight at 4 °C. Washing and elution were carried out as described under GFP-human MAU-2 immunoprecipitation.

### Flow cytometry.

HeLa cells were subjected to siRNA-mediated gene knockdown, synchronization in G2/M by nocodazole, and released to progress into the cell cycle as above. Aliquots were collected at 0 and 3.5 h, cells were washed in PBS, and then fixed in [70% ethanol, 30% serum-free DMEM]. For analysis, cells were re-hydrated and permeabilized in PBS [0.1% Triton X-100], treated with RNase A (0.2 mg/ml), and stained with propidium idodide (0.02 mg/ml). The samples were analyzed using a FACSCalibur ^TM^ fluorescence-activated cell sorter (BD Biosciences, San Diego, California, United States).

### RNAi and visualization of chromosome segregation defects in *C. elegans*

Standard methods were used for maintaining and manipulating C. elegans [ 65]. A histone::GFP strain (F54E12.4::GFP = H2B::GFP; see [ 66]) was used to visualize chromosome segregations. Double-stranded RNA designed to interfere with *scc-3, mau-2,* or *pqn-85* function was generated by in vitro transcription of a gel-purified PCR product flanked by T7 and T3 promoter sequences as described [ 67]. The RNA was injected into the gonad of young histone::GFP worms and the progeny from injected worms were assayed at 20 °C. The forward (F) and reverse (R) primers used were as follows: *scc-3*F *:* 5′ – AATTAACCCTCACTAAAGGACAGCGACTTTTTGCGCTAT – 3′; *scc-3*R: 5′ – TAATACGACTCACTATAGGTCGATGAAGAACGTCGTGAG – 3′; *mau-2*F: 5′ - AATTAACCCTCACTAAAGGAGCGCCTTTCAAAAATCAAA – 3′; *mau-2*R: 5′ – TAATACGACTCACTATAGGCGCCAAAAATGTGAAAACTG – 3′; *pqn-85*F: 5′ – AATTAACCCTCACTAAAGGCCGCCAATAATTCAAGCAGT – 3′; *pqn-85*R: 5′ – TAATACGACTCACTATAGGTGTGACCCAAAGAAATGCTG – 3′. All of the above PCR reactions were carried out using C. elegans genomic DNA as a template. Worms were dissected in water to remove early stage eggs from the gonad 48 h after injection of RNA. The released eggs were transferred to a fresh agarose pad (2%) and the histone::GFP was visualized using a Zeiss Axiophot microscope.

### Antisense morpholino oligonucleotide-mediated inhibition of mRNA translation in X. tropicalis embryos.

X. tropicalis priming and fertilizations were carried out as described [ 68]. Antisense morpholino oligonucleotides ( AGCTGTAGGATGGTGGTATCTATGC and CGTAGCCGCCATCACTGACATCAAC designed to inhibit translation of *X. tropicalis Nipbl* and *KIAA0892* mRNAs, respectively) were modified by addition of lissamine fluorochromes. One-cell embryos were injected with 10 ng of test or control (GeneTools standard control) morpholino in 1 nl H _2_O. Embryos were observed, photographed, and fixed in 4% formaldehyde at gastrula (stages 10–12) and tailbud (stages 24–28).

### Mutation screening of *KIAA0892*

We screened a panel of 18 CdLS patients that had not previously been found to have mutations in *NIPBL* for mutations in *KIAA0892,* the human ortholog of *C. elegans mau-2.* Exons and immediately neighboring intronic sequences were amplified using the primers detailed in Table 1. Mutation screening was carried out by direct sequencing using the MegaBACE ET system (Amersham, Little Chalfont, United Kingdom) and, where exons were suitably small, by single-strand conformation polymorphism (SSCP)—heteroduplex analysis using standard protocols. In the latter case we denatured PCR products, size fractionated them in 1x MDE gel (BioWhittaker, Rockland, Maryland, United States) containing 5% glycerol and 0.6% TBE buffer at 300 V for ˜ 20 h (depending on fragment size), and visualized them by silver staining. Any samples that had differences relative to an unaffected control were subsequently sequenced.

**Table 1:**
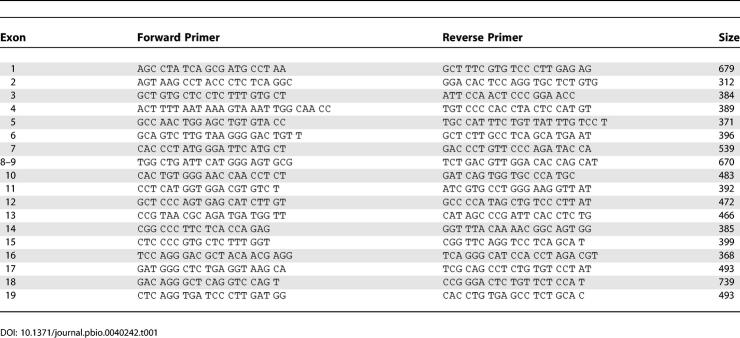
Primers Used to Amplify Exons of the *KIAA0892* Gene

## Supporting Information

### Accession Numbers

The protein sequences of various members of the metazoan MAU-2 family used for sequence comparisons had accession numbers listed at the US National Center for Biotechnology Information (NCBI) Web site ( http://www.ncbi.nlm.nih.gov) as follows: human KIAA0892 protein (Q9Y6X3), D. melanogaster CG4203-PA protein (Q9VFC0), S. purpuratus (XP_784063), X. tropicalis (NP_001015927), and chick (XP_425908). Note that the human sequence Q9Y6X3 contains eight incorrectly predicted amino acids at the N-terminal end, which were omitted for the purpose of sequence comparison, and the chick sequence was amended to remove sequence from two incorrectly predicted exons.

The Online Mendelian Inheritance in Man (OMIM) ( http://www.ncbi.nlm.nih.gov/OMIM) accession number for Cornelia de Lange syndrome is 122470.

## Abbreviations

GFP: green fluorescent protein
PSCS: precocious sister chromatid separation
RNAi: RNA interference
siRNA: short interfering RNA
